# Impact of an Educational Programme in Primary Care for mothers With Infant Colic Babies: Quasi-Experimental Study

**DOI:** 10.1177/00099228261423662

**Published:** 2026-03-07

**Authors:** María del Mar Martínez-Lentisco, Sonia Carreño-Segura, Felipe León-Morillas, Juan Manuel García-Torrecillas

**Affiliations:** 1 Department of Nursing, Physiotherapy and Medicine, University of Almería, La Cañada de San Urbano, Spain; 2 Health District Almeria, Andalusian Health Service, Almería, Spain; 3 Department of Health Sciences, Faculty of Health Sciences, University of Jaén, Jaén, Spain; 4 Clinical-Epidemiological Research Area, Torrecárdenas University Hospital, Almería, Spain; 5 CIBER de Epidemiología y Salud Pública (CIBERESP), Madrid, Spain; 6 Instituto de Investigación Biosanitaria Ibs, Granada, Spain

**Keywords:** colic, physical therapy modalities, primary health care, health education, quality of health care

## Abstract

This quasi-experimental study assessed the impact of a postpartum educational programme on health care utilization and maternal stress in mothers of infants with colic. A total of 84 mothers took part in 2 individual sessions, 15 days apart. Maternal stress was measured at baseline and again at 3 months using Perceived Stress Questionnaire; colic severity was evaluated with Infant Colic Questionnaire Severity. Analyses included the Wilcoxon test, repeated-measures analysis and Spearman correlation. Maternal stress levels were not significantly related to colic severity, feeding type, childbirth type or the number of paediatric or emergency visits. However, we observed a statistically significant reduction in health care visits for excessive crying during the intervention period (*P* < .01). Although the programme did not reduce maternal stress directly, mothers rated it highly satisfactory, and it effectively lowered unnecessary health care use. Early postpartum education and support programmes may enhance care quality and optimize health care resource utilization in primary care.

Trial Registration: Clinicaltrials.gov NCT 03326297

## Introduction

The first months of life, the crying and irritability of infant colic causes a high number of repeated consultations and consumption of health care resources.^1,2^ Maternal education programmes are a tool to improve maternal and newborn health.^3^ The postpartum period is overlooked as a stage of risk for the mother and a period in which educational intervention is needed to promote the autonomy of mothers and to use education as a means of promoting health in the mother-child dyad.^4^

Risk factors for perinatal depression include stressful life events as well as the type of medical coverage.^5^ Experiencing psychosocial distress during pregnancy has been associated with a higher risk of having an infant with colic.^6^ Moreover, mothers of infants diagnosed with colic report significantly higher levels of stress than those whose infants do not present this condition.^7^ The presence of stress has an influence on maternal emotional state^8^ and motherhood can influence mental health by contributing to increased stress during this period.^9^ Infant colic is recognized as a multifactorial condition, where crying episodes are not solely attributable to gastrointestinal discomfort but may reflect a broader interplay of perinatal, behavioural and psychosocial influences.^10^

However, despite the existence of these educational strategies internationally, the implementation of postnatal support in our specific setting is limited, currently in our community these programmes are only carried out before the birth through group interventions with the pregnant woman. There is a lack of care, prevention and health promotion in the postpartum period in women, which can have a negative influence on the first months of childbearing and on the learning and real monitoring of the baby’s first care.

The aim of this study was to analyze whether a structured health education programme reduces maternal stress and health care utilization related to excessive infant crying. We also examined the potential relationship between maternal stress and the frequency of visits to pediatric primary care and emergency services. In addition, we assessed whether maternal stress is associated with the severity of infant colic, type of childbirth and type of feeding.

## Methods

Quasi-experimental pre-post study without a control group with a sample of mothers with babies with excessive crying diagnosed by their paediatrician as infant colic the absence of organic pathology justifying the same. A sample size of 84 participants was determined, considering a confidence level of 95%, a statistical power of 80%, an expected effect of 0.4 units on the maternal stress scale and a variability of 1.2 units. A sample size calculator was used for before-after mean comparison studies. Participant selection was performed using a non-randomized sequential sampling method. We have adhered to relevant EQUATOR guidelines and used the Trend guidelines. Data collection was carried out between November 2017 and November 2019. Mothers were invited to participate in the study in the Almeria Health District (Spain).

### Participants

Mothers of healthy infants diagnosed with infant colic by their paediatrician, who presented frequent crying episodes and adequate weight gain. Inclusion criteria: Mothers with an infant diagnosed with infant colic by paediatrician; assisted in primary care centre; born at 37 or more weeks of gestation; and providing informed consent to participate. Exclusion criteria: Mothers diagnosed with mental disorder; Currently under pharmacological treatment for mental health conditions (eg, antidepressants, anxiolytics); inability to communicate in Spanish; or cognitive or language barriers that could interfere with participation in the program or completion of questionnaires.

### Intervention

The intervention was performed on 2 occasions, the first after referral by their paediatrician and acceptance of their participation in the study (T0) and a second after 15 days, for about 60 minutes each session. A health education programme was carried out by the primary care physiotherapist, and some babies also received osteopathic manual therapy treatment from the physiotherapist. Participants were blinded to the specific manual therapy technique. To achieve this, a sham (simulated) manual protocol was implemented for the control group. Since the mothers were not trained in manual therapy, they could not distinguish between the specific osteopathic therapeutic maneuver and the simulated manual contact, ensuring effective blinding. This programme consisted of advice on how to manage the baby’s crying crises, advice related to the effective establishment of breastfeeding and attention to the baby’s hunger signals, advice on attachment and manual-postural contact with the baby. As well as resolving any doubts about parenting that they may have in the consultation room. All sessions were conducted by the same physiotherapist researcher, ensuring consistency in the delivery of the educational programme across all participants. They were treated in an empathetic manner, always respecting their preferences and expectations, as well as respecting their privacy and creating a climate of trust and security, with a maximum level of confidentiality.

Intervention was carried out individually in the paediatrician’s office or in the physiotherapy office, depending on the availability of space. They came to the consultation with their baby and could be accompanied by the adults involved in the child’s upbringing.

### Data Collection

In the first consultation (T0), they were provided with detailed information about the study, given informed consent, data related to child rearing were obtained, such as type of feeding, type of birth, demand for care so far from the paediatrician and/or the emergency department for excessive crying, use of products independently to soothe the baby and internet consultations on child rearing.

The severity of infant crying was assessed using the Infant Colic Severity Questionnaire (ICSQ)^11^ both at baseline (T0) and at 3 months of age (T1), assesses the severity of colic episodes as perceived by the caregiver. The total score ranges from 36 to 100, with higher scores indicating greater severity of infant colic, scores below 50 suggest symptoms of immaturity and are not considered indicative of infant colic. Scores between 51 and 75 reflect moderate colic, while scores above 75 indicate severe colic with a Cronbach’s alpha of 0.72. Satisfaction with the intervention was assessed through an ad hoc questionnaire (not satisfied, partially satisfied, fully satisfied) (T1) and the perceived resolution of the infant’s crying condition through a questionnaire (not resolved, partially resolved, fully resolved) (T1).

The Perceived Stress Questionnaire (PSQ)^12^ was used to assess stress. The questionnaire consists of 30 items that assess stress in the last 30 days, resulting in an index between 0 and 1, where 0 is the lowest stress and 1 the highest, with a Cronbach’s alpha of 0.82. Mothers were assessed for stress on 2 occasions, at baseline, after diagnosis by their paediatrician of babies with excessive crying (T0) and at 3 months of age (T1).

### Data Analysis

A descriptive statistical analysis was performed where continuous variables were expressed as mean and standard deviation and categorical variables as a table of frequencies and percentages. To determine the differences between the demand for care before and during the intervention, the Wilcoxon test was used. To analyse whether there are differences in stress before and after the intervention, repeated measures analysis was used. Spearman’s correlation was used to determine the correlation between quantitative variables. Statistical analysis was performed using Stata version 12 (StataCorp LLC, College Station, TX, USA).

### Ethical Considerations

The study was conducted in accordance with the Declaration of Helsinki and approved by the Research Commission.

## Results

Table 1 presents the descriptive characteristics of the sample. At the beginning of the intervention, perceived stress levels were moderate and showed no significant relationship with other variables, such as feeding type or type of childbirth. Satisfaction with the educational programme was high (84%). In addition, results indicated that a significant proportion of mothers consulted the internet for parenting questions. Regarding birth order, 47.1% of the participants were first-time mothers, 41.7% had a second child and 10.7% had a third.

**Table 1.** Stress and Relationship With Socio-Demographic Variables.

Perceived stress questionnaire	Median (IQR)	N (%)	*P* value
**Baseline**	0.41 (0.10)	0.10	
**3 months**	0.38 (0.21)		
**Variables**			
**Childbirth type**	Spontaneous vaginal	37 (44.04)	.70^a^
	Planned caesarean	11 (13.09)	
	Instrumental vaginal	25 (29.76)	
	Unplanned caesarean	11 (13.09)	
**Type of feeding**	Maternal	32 (38.09)	.24^a^
	Artificial	26 (30.95)	
	Mixed	26 (30.95)	
**Colic relief products**	No	10 (11.90)	.28^b^
	Yes	74 (88.09)	
**Search for information on the web**	No	31 (36.90)	.62^b^
	Yes	53 (63.09)	
**Satisfaction with the intervention**	Partially	13 (15.47)	.63^c^
	Fully	71 (84.52)	

a Kruskal-Wallis.

b Mann-Whitney *U*.

c Wilcoxon.

As shown in Table 2, the correlation between paediatric visits and maternal stress was weak and negative and did not reach statistical significance. Similarly, the correlation for emergency department visits was weak, negative and non-significant.

**Table 2.** Relationship Between Stress and Number of Visits to the Paediatrician and Emergency Department.

Variable	Mean (SD)	Spearman’s rho (*P*-value)	Wilcoxon test
**Paediatrician visits**			
Visits before intervention	1.18 (0.73)	–0.18 (0.08)	
Visits during follow-up	0.35 (0.67)		*P* < .01
**Emergency department visits**			
Visits before intervention	0.61 (0.88)	–0.20 (0.06)	
Visits during follow-up	0.18 (0.49)		*P* < .01

Statistically significant differences were observed in the frequency of paediatric and emergency department visits between the pre-intervention and intervention periods. These visits correspond specifically to those reported for infant colic during the 3-month follow-up. As indicated in Table 3, no significant relationship was found between the severity of infant colic (categorized as no colic, moderate or severe) and the frequency of paediatric or emergency visits.

**Table 3.** Severity of Colic and Visits to the Paediatrician and Emergency Department for Infant Colic.

Severity level (ICSQ)	Baseline N (%)	Paediatrician visits mean (SD)	Emergency visits mean (SD)	3 months N (%)
Normal	24 (28.57)	0.95 (0.80)	0.58 (0.88)	59 (70.24)
Moderate	58 (69.05)	1.08 (0.78)	0.50 (0.84)	24 (28.57)
Severe	2 (2.38)	1.00 (0.00)	0.50 (0.70)	1 (1.19)
*P*-value		.78	.95	

Abbreviation: ICSQ, Infant Colic Severity Questionnaire.

**Figure 1.**
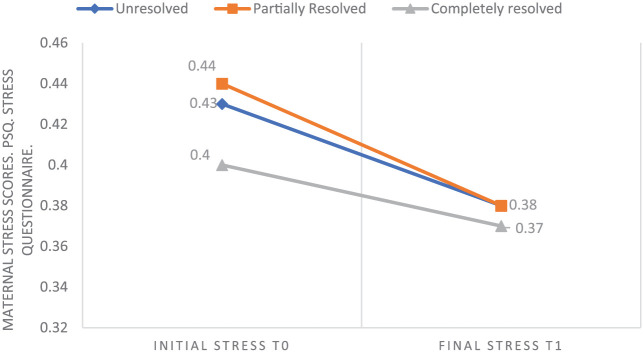
Evolution of maternal stress up to 3 months of age. Results of the PSQ maternal stress questionnaire at the beginning of the intervention and 3 months after the baby’s birth. Maternal stress decreases independently of the perceived resolution of the crying episode.

No significant association was detected between stress levels and the severity of crying episodes. Correlations were weak both at baseline (T0) (Spearman rho = 0.09, *P* = .37) and after the educational programme (T1) (Spearman rho = 0.24, *P* = .11). Regarding the relationship between the perceived resolution of the crying episode and maternal stress, repeated-measures analysis indicated that stress levels did not vary significantly over time. Similarly, the interaction between time and the perceived resolution group was not statistically significant. As illustrated in Figure 1, maternal stress levels showed a non-significant decreasing trend during the follow-up period (up to 3 months of age), regardless of whether mothers considered the colic to be unresolved, partially resolved or completely resolved.

## Discussion

This study investigates the relationship between maternal stress, infant crying spells and the demand for medical care. Inconsolable crying is a significant cause of parental distress and is associated with a deficit in maternal efficacy, which increases the risk of mental symptoms in the postpartum period.^13^ It has previously been observed that mothers of infants with colic show more stress than mothers of infants without colic.^7^ However, in our study, the reported stress levels were average. We observed that a high percentage of parents had to consult the internet for parenting questions; therefore, we agree with Ellet and Swenson on the need for support for families of babies with excessive crying.^14^ The educational programme used in our study for postpartum care can be considered a key component of social support for the mother, acting, as suggested by Alexander et al,^15^ as a preventive factor in the baby’s crying patterns.

Although we cannot affirm that maternal stress is directly related to the severity of crying spells in our sample, we observed significant outcomes regarding health care use. Consistent with the findings of Barr et al,^16^ the number of visits made to health services to seek support decreased in a statistically significant way. It is noteworthy that while Barr et al demonstrated this effect through a broad public health campaign, our results confirm that a targeted, in-person educational intervention in Primary Care achieves a similar reduction in health care demand.

Regarding the association between stress and colic severity, our results differ from those of de Kruijff et al,^7^ who found higher stress in mothers of infants with colic. A possible explanation for this discrepancy is that the educational intervention in our study may have empowered mothers with coping mechanisms, effectively dissociating their perceived stress from the objective severity of the infant’s crying.

These findings support the idea that the etiology of infantile colic is complex and that perinatal conditions and maternal history play an important role.^10^ By focusing on maternal stress and its connection to infantile colic, our study supports this view. Our findings point to the need for a more comprehensive approach, one that considers both the psychological and health care needs of mothers. To reduce colic symptoms and improve the well-being of both mothers and their infants, interventions must identify and address maternal factors, such as stress and mental health. Therefore, future studies and clinical procedures should take a holistic approach that incorporates the mental and physical well-being of the mother. In addition, future research should consider incorporating qualitative methods to explore mothers’ self-perceived stressors and their specific experiences with the educational programme in greater depth.

The results of our study show a high level of satisfaction with the intervention, making it an effective tool in the promotion of parenting skills, as suggested by the lines of intervention of the health system.^17^ This demonstrates the relevance of establishing early support in families. While prenatal education programmes in our environment have generally proven effective for childbirth preparation, they often lack specific strategies for managing infant colic, which typically manifests weeks later. This temporal gap highlights the necessity of the postpartum educational intervention presented herein, which provides support exactly when the symptoms and parental stress peak, filling a void that prenatal education cannot fully address. We consider that holistic intervention is necessary in the mother-baby dyad after childbirth, by including strategies for the effective establishment of breastfeeding that promote the mother’s self-efficacy and mental health.^18^

The possible lack of satisfaction with parenting, derived from fatigue, the feeling of lack of self-efficacy in caring for the baby, and influenced by societal and cultural factors, can plunge mothers into situations of grief that give rise to a variety of emotional symptoms. To avoid this, a maternal education programme could be favourable as it brings physical, psychological and social benefits for both mothers and baby.^1^ We therefore stress that care for the mother in these cases is as necessary as care for the baby, agreeing with authors such as Lorén-Guerrero et al,^19^ who emphasize the importance of health care with the support of professionals to reduce the psychological impact.

Similarly, our results are in line with those of Al Qahtani and Ahmed, who showed that educational programmes for the mother are favourable in reducing crying symptoms.^20^ Emotional support, care and information dedicated to parenting and breastfeeding are necessary and favour a better adaptation during this period,^21^ as well as favouring bonding—which can be affected in babies with behavioural problems^22^ and maternal sensitivity.^23^ Our results show no relationship between the type of infant feeding and maternal stress, although it may seem a priori that exclusive breastfeeding is a stress factor for the mother. Prior experience in caregiving did not appear to influence satisfaction with the programme or the demand for health care services. These findings suggest that parenting experience alone may not be sufficient to mitigate stress or reduce health care utilization in this context.

### Limitations

Our study is subject to certain limitations as indicated below, we cannot evaluate the application of the recommendations at home, what we can access is the verification in the consultation that they have understood them correctly, as well as the offer to resolve doubts, if needed, although the participants are satisfied with the empowerment in the management of crying crises, with the improvement in the feeling of self-efficacy and with the non-imposing therapeutic connection The questionnaire to evaluate stress is not specific to postpartum, although it evaluates the perception of stress in the last month. It would be interesting to compare the results with a group without such intervention.

### Implications

The puerperium is considered a vital moment that involves a great deal of adaptation and adjustment by the mother and the family group to the arrival of a new member, which is why we consider it necessary to include an educational programme in this period to help resolve doubts and avoid the use of health services in the face of the difficulties inherent in childbearing. We believe it is essential for the health system to resolve the doubts generated in childbirth, thus ensuring reliable information provided by up-to-date professionals on the subject, since improving birth conditions and neonatal care, as well as promoting breastfeeding, form part of the national health system’s childbirth and birth care strategies.^24^

A physiotherapist integrated in the primary care team can bring about changes in the frequency of consultations and improve mothers’ autonomous management, with the possible impact on the efficiency of the system. It is a scalable, multidisciplinary and low-cost alternative.

## Conclusion

A postpartum health education programme, implemented within primary care, contributed to a significant reduction in paediatric and emergency department visits related to excessive crying. Maternal stress was not influenced by factors such as the severity of infant colic, the type of feeding or type of delivery. No observed relationship between maternal stress and the independent use of soothing products or consultations outside the health care system.

Although these programs did not reduce maternal stress directly, they proved valuable in lowering unnecessary health care demand linked to infant colic. These findings highlight the potential benefits of early health education interventions in optimizing health care use and offer much-needed assistance to families.

## Author Contributions

**María del Mar Martínez-Lentisco:** Conceptualization; methodology; software; validation; formal analysis; data curation; writing original draft preparation; writing – review and editing; visualization; project administration; supervision; funding acquisition. **Juan Manuel García-Torrecillas:** Conceptualization; methodology; software; validation; data curation; writing – review and editing; visualization; supervision; project administration. **Sonia Carreño-Segura:** Conceptualization; validation; writing – review and editing; visualization; supervision. **Felipe León-Morillas:** Conceptualization; visualization; validation; writing – review and editing; methodology, supervision; formal analysis. All authors have read and agreed to the published version of the manuscript.
